# Platelet Vascular Endothelial Growth Factor is a Potential Mediator of Transfusion-Related Acute Lung Injury

**DOI:** 10.4172/2161-105X.1000212

**Published:** 2014-10-20

**Authors:** James P Maloney, Daniel R Ambruso, Norbert F Voelkel, Christopher C Silliman

**Affiliations:** 1 Departments of Pulmonary Sciences and Critical Care Medicine, University of Colorado at Denver, USA; 2 Department of Medicine, University of Colorado at Denver, USA; 3 Department of Pediatrics, University of Colorado at Denver, USA; 4 Department of Pathology, University of Colorado at Denver, USA; 5 Department of Surgery, University of Colorado at Denver, USA; 6 University of Colorado School of Medicine; the Center for Cancer and Blood Disorders, Children’s Hospital Colorado, USA; 7 Department of Internal Medicine, Commonwealth University of Virginia, USA; 8 Bonfils Blood Center, University of Colorado at Denver, USA

**Keywords:** Apheresis, Platelets, Storage, Vascular endothelial growth factor, Lung injury, Leukocytes, Transfusion

## Abstract

**Objective:**

The occurrence of non-hemolytic transfusion reactions is highest with platelet and plasma administration. Some of these reactions are characterized by endothelial leak, especially transfusion related acute lung injury (TRALI). Elevated concentrations of inflammatory mediators secreted by contaminating leukocytes during blood product storage may contribute to such reactions, but platelet-secreted mediators may also contribute. We hypothesized that platelet storage leads to accumulation of the endothelial permeability mediator vascular endothelial growth factor (VEGF), and that intravascular administration of exogenous VEGF leads to extensive binding to its lung receptors.

**Methods:**

Single donor, leukocyte-reduced apheresis platelet units were sampled over 5 days of storage. VEGF protein content of the centrifuged supernatant was determined by ELISA, and the potential contribution of VEGF from contaminating leukocytes was quantified. Isolated-perfused rat lungs were used to study the uptake of radiolabeled VEGF administered intravascularly, and the effect of unlabeled VEGF on lung leak.

**Results:**

There was a time-dependent release of VEGF into the plasma fraction of the platelet concentrates (62 ± 9 pg/ml on day one, 149 ± 23 pg/ml on day 5; mean ± SEM, p<0.01, n=8) and a contribution by contaminating leukocytes was excluded. Exogenous 125I-VEGF bound avidly and specifically to the lung vasculature, and unlabeled VEGF in the lung perfusate caused vascular leak.

**Conclusion:**

Rising concentrations of VEGF occur during storage of single donor platelet concentrates due to platelet secretion or disintegration, but not due to leukocyte contamination. Exogenous VEGF at these concentrations rapidly binds to its receptors in the lung vessels. At higher VEGF concentrations, VEGF causes vascular leak in uninjured lungs. These data provide further evidence that VEGF may contribute to the increased lung permeability seen in TRALI associated with platelet products.

## Introduction

The association of blood product transfusion with a variety of systemic reactions is well described. The most common of these reactions are the non-hemolytic transfusion reactions which occur most often with platelets, particularly with units stored for longer periods, and appear to be related most often to soluble substances in the transfused plasma [1]. These disorders include a reaction characterized by non-cardiogenic pulmonary edema known as transfusion-related acute lung injury (TRALI) [2].

The storage of human platelets is associated with time-dependent increases in soluble pro-inflammatory cytokines such as interleukin-6 (IL-6), IL-8, IL-1β, tumor necrosis factor-α, and vascular endothelial growth factor (VEGF) [3–5]. Contaminating leukocytes may be the predominant source of elevations in cytokines which have been linked to some transfusion reactions, but such elevations are detectable in only a minority of platelet units implicated in non-hemolytic transfusion reactions [6]. The use of pre-storage leukocyte-depleted platelet units (via either filtration or by more selective apheresis techniques) resulted in fewer transfusion reactions in several but not all studies, highlighting that leukocytes explain only some reactions [7,8]. Platelets themselves likely contribute to these reactions as some 15–20% of platelets become activated during storage with subsequent degranulation and release of mediators such as soluble CD40 ligand (sCD40L) [9]. As single donor apheresis platelet units have less leukocyte contamination than pooled (whole blood) units, they are useful for studying mediator release during storage.

TRALI is clinically identical to acute lung injury (ALI) except that most patients with TRALI survive with recovery in 48–72 hours [10]. TRALI has a temporal association with transfusion and may occur following transfusion of a small volume of products [11]. Various models have been proposed for the pathophysiology of TRALI based upon human data and animal work [9,12,13]. These models have focused on the neutrophil, soluble activators of leukocytes (such as sCD40L), and MHC or anti-granulocyte antibodies as the primary effectors of ALI [14]. Most cases of non-antibody mediated TRALI are associated with administration of products with longer storage times [15]. Interestingly, the supernatant from day 5 leuko-reduced human platelet products causes lung injury when administered intravascularly to rats pre-treated with endotoxin (thus activating lung endothelium and circulating leukocytes), whereas this does not occur with day 0 plasma or when animals are not pre-treated with endotoxin [1]. These data fit the “two-hit” model of TRALI, where activation of leukocytes or endothelium is needed [15,16]. Only one of the substances that increase during routine platelet storage is a direct acting permeability agent - VEGF [17] which led us to postulate that VEGF may be an important mediator of TRALI. In this scenario, VEGF released during platelet storage would bind avidly to its receptors in the lung vasculature during transfusion, contributing to increases in lung permeability - particularly if the vasculature was previously injured/activated.

We hypothesized that VEGF concentrations increase in the plasma of leuko-reduced platelet suspensions over the course of storage due to platelet activation and release (rather than release from contaminating leukocytes), and that VEGF avidly binds to its lung receptors at VEGF concentrations typical of stored platelet supernatants.

## Methods

GeneralAll plastics, solutions, chemicals, and buffers were from Sigma (St. Louis, MO) unless otherwise stated.

### Platelet isolation

Apheresis platelet concentrates were obtained from eight healthy adult volunteers (each donated one unit) after informed consent was obtained as above. COBE Spectra cell separation system equipped with the LRS^®^ system for producing platelet concentrates containing very low levels of residual leukocytes was employed [18]. Platelets were stored at 20–24°C with constant agitation under standard conditions per AABB criteria [19]. Aliquots of platelet units were also removed for cell lysis measurements on day 1. Samples of 15–60 ml were withdrawn on the day after collection (day 1) through day 5 via couplers and sterile technique. These samples were then centrifuged at 180× g for 15 minutes at 22°C, the supernatant was then removed and centrifuged at 2000× g for 10 minutes. Final supernatants were then frozen at −70°C until later analysis. The original platelet units contained 1.29 ± 0.17 × 10^9^ platelets/ml and 201 ± 320 total leukocytes/ml (mean ± SEM) in volumes averaging 267 ± 19 ml (n = 8, mean ± SEM).

### Human leukocyte isolation

Leukocytes were separated from heparinized (1U/ml) whole blood from healthy adult donors after obtaining informed consent under the auspices of a protocol approved by the Colorado Multi-Institutional Review Board. Neutrophils were isolated by standard techniques including dextran sedimentation, ficoll hypaque gradient centrifugation and hypotonic lysis of contaminating red blood cells and the granulocytes were counted employing a blood cell counter as previously described [20].

### Cell lysis

Freshly isolated cells were washed in PBS (in endotoxin-free H20, pH 7.4) then pelleted at 180× g for 10 minutes at 22°C and lysed. Platelet pellets containing 1011 cells were disrupted by incubation in 5 ml of lysis buffer [10 mmol/L Hepes, 137 mmol/L NaCl, 2.9 mmol/L KCl, 12 mmol/L NaHCO_3_, 2% NP-40, 25 mmol/L deoxycholic acid (1%), 7 mmol/L SDS (0.2%)] with protease inhibitors (PMSF 0.1 mmol/L, leupeptin 20 µg/ml, and aprotinin 20 µg/ml) at 4°C for 30 minutes. As these resuspended platelets had contaminating leukocyte concentrations of approximately 2 ×10^4^ leukocytes/ml, leukocytes isolated from whole blood were similarly lysed at 2 × 10^5^ neutrophils/ml and 2 × 10^5^ mononuclear cells/ml. These leukocyte concentrations were chosen as they were 10-fold higher than those contaminating our apheresis platelet concentrates. Lysates were then centrifuged at 14,000× g in a microcentrifuge at 4°C for 10 minutes and the supernatants were aliquotted and frozen at −70°C for later analysis. Protein concentration was measured by a modified Lowry assay (Sigma).

### ELISA for VEGF

The concentration of VEGF ([VEGF]) was determined in duplicate with a commercial ELISA (R&D Systems, Inc.; Minneapolis, MN). This assay is sensitive to 9 pg/ml (0.2 pM) VEGF. Optical density (450 nm) was measured on a plate reader and corrected for background.

### Iodogenation of VEGF

5 mcg of carrier-free human VEGF165 (R&D Systems, Inc.) was iodinated with 1mCi of Na125I as described [21]. Labeled VEGF was then separated from free 125I by passage over a Sephadex G25M PD-10 and Hi-Trap Heparin columns (Pharmacia, Alameda, CA). This yielded a solution that contained 99% of counts in the bound (125I-VEGF) form based on precipitation with cold 10% TCA. Specific activity was 1909 Ci/mMole. Purity was confirmed by migration of a single 22 kDa band (monomer) on reducing SDS-PAGE autoradiography. 125I-VEGF enhanced 3H-thymidine incorporation in umbilical vein endothelial cells and thus remained bioactive.

### Isolated perfused rat lungs (IPRL)

The protocol was previously approved by the Univ. of Colorado animal use committee. The IPRL procedure was performed as per published reports [13]. Pathogen-free male Sprague-Dawley rats of 250–350 grams were obtained from Jackson Labs (Bar Harbor, Maine). After i.p. injection with sodium pentobarbital, the animals were tracheostomized and ventilated with 6 ml/kg tidal volume, rate 40, peep 3 with humidified/warmed 21% O_2_, 5% CO_2_, balance N_2_ on a Harvard apparatus. After median sternotomy and injection of heparin into the right ventricle, the pulmonary outflow tract was cannulated and tied off, followed by cannulation of the left ventricle, whereupon perfusion was begun with a peristaltic pump. The trachea, lungs, and heart were dissected free and suspended at 37 °C in a humidified chamber. After a pre-equilibration period of 30 minutes the perfusion circuit was closed and target perfusion of 0.03ml/gram rat weight/minute was maintained for 15 minutes before experiments. Pulmonary artery pressure was measured continuously. Perfusate [Earle’s balanced salt’s solution (EBS) with 4% Ficoll, pH 7.4] was maintained at 37°C.

### 125I-VEGF Binding to Isolated Perfused Rat Lung (IPRL) microvasculature

To test the potential binding of infused VEGF to its receptors in the lung vasculature, experiments were performed using IPRL with 125IVEGF added to the perfusate. Specific 125I-VEGF binding was assessed in four experimental conditions: 1) addition of 125I-VEGF preincubated for 30 min with 10 ul of PBS vehicle; 2) “Cold VEGF” - perfusate containing 30ng/ml of unlabeled VEGF (1.3 nM, > 1000X concentration of 125I-VEGF) was circulated for 15 min before addition of 125I-VEGF (that had been pre-incubated for 30 min with 10 ul of PBS vehicle); 3) perfusate was circulated for 15 min before addition of 125I-VEGF that had been pre-incubated for 30 minutes with 0.5 mcg of anti-VEGF IgG antibody 4.6.1 (Genentech, South San Francisco, CA; in 10 ul of PBS vehicle) or 4) perfusate was circulated for 15 min before addition of 125I-VEGF that had been pre-incubated for 30 minutes with 0.5 mcg of isotype control anti-CMV IgG min (in 10 ul of PBS vehicle). At time zero in each experiment, 0.1 uCi (154,000 counts per minute, cpm) of 125I-VEGF was added to the perfusate (after the antibody or PBS incubations as above). The final concentration of 125I-VEGF of 1 pM (23 pg/ml) is similar to VEGF concentrations in the platelet plasma fractions. After 15 minutes the 125I-VEGF-containing perfusate was switched to 50 ml of nonradioactive and non-recirculating EBS/4% BSA to remove unbound 125I-VEGF. This resulted in less than 250 cpm/ml in perfusate exiting the left ventricle at completion of the wash. Perfusion was then stopped, the lungs and heart were dissected free, blotted dry, weighed, and placed in vials for gamma-counting. Controls were done on the same day, with an n ≥ 3 for all groups. Lung vascular leak was not determined in these experiments as we could not discern between 125I-albumin and 125I-VEGF by radioactivity measurements, and as the long half-life of 125Iodine precluded using other methods in our lab (determination of wet-to-dry ratios by evaporative drying of lungs requires intentional venting of radioactivity to ambient air - precluded by our institutional radiation safety policy, and Evans Blue dye leak techniques were not feasible due to the prospect of contamination and difficult cleaning of heavy utilized tissue grinding equipment).

### Assessment of vascular leak in isolated perfused rat lungs (IPRL) by 125i-albumin translocation

Uninjured isolated perfused rat lungs (IPRL) were studied with 125I-albumin added to the perfusate to determine the lung vascular leak caused by unlabeled VEGF (250 pM, or 5,750 pg/ml in PBS) compared to PBS vehicle. The 125I-albumin leak IPRL model was performed as published by our lab [22]. This concentration of VEGF was chosen as it was between those noted in vivo to cause vascular leak of uninjured rat lungs (7 nM) and porcine coronary venules (100 pM) [23,24]. These experiments were performed before the levels of VEGF in stored platelets were known, and were not repeated at lower VEGF concentrations due to limited funds. 125I-VEGF was not used in these experiments. Leak was determined by counts per minute (CPM) of radioactivity present in each gram of wet lung tissue. Perfusion with 125I-albumin was done for 15 minutes after VEGF addition.

### Statistical Analysis

Linear regression was used to determine for VEGF concentration ([VEGF]) by ELISA. A nonparametric student’s T-test was used to compare day 1 and other day [VEGF] in the apheresis units (paired) and differences in radioactivity levels in IPRL between treatment groups (unpaired). Group data are presented as mean ± SEM. [VEGF] was determined by linear regression from a standard curve. All analyses were performed with GraphPad software (San Diego, CA) and all were two-tailed. Significance was defined as p < .05.

## Results

### VEGF is released from platelets as a function of storage time

**Figure 1:**
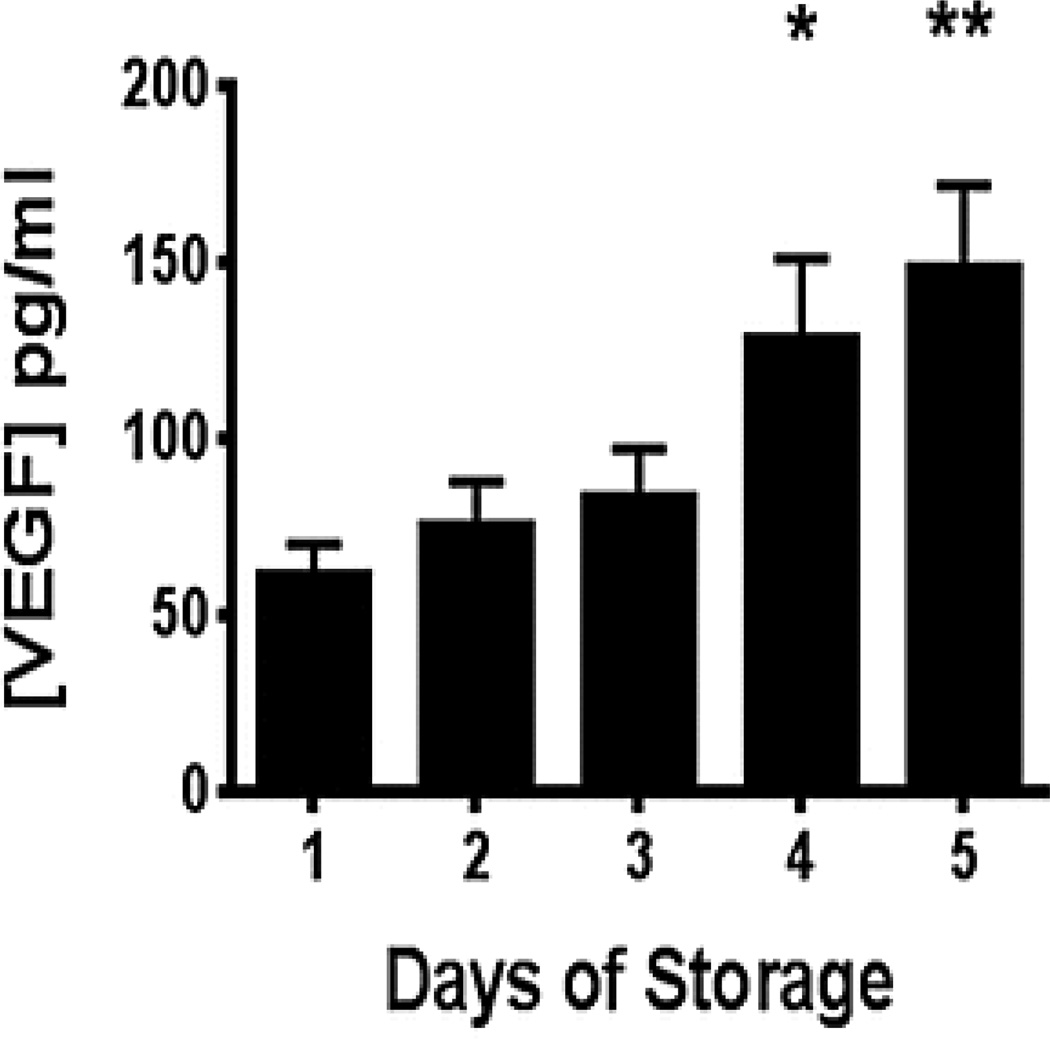
Release of VEGF into the plasma fraction of stored leuko-reduced apheresis platelet units over 5 days (N = 8). Aliquots of platelets were centrifuged and supernatant VEGF levels were quantified by ELISA. *p < .05 vs. day 1 value, **p < .001 vs. day 1 value. Results are depicted as group mean ± SEM

Figure 1 depicts the release of VEGF into the supernatant of apheresis platelet units during routine storage (5 days). Data are averaged from individual donors (n=8). Each of the donor platelet units released some amount of VEGF over time. There was a significant difference in the amount of VEGF released when the individual donors (n = 8) were compared on day 1 (62 ± 9 pg/ml; mean ± SEM) versus days 4 (129 ± 51 pg/ml; p = 0.023) and day 5 (149 ± 52 pg/ml; mean ± SEM, p = 0.008). These VEGF concentrations are similar to those measured in one other study of apheresis platelet unit storage but lower than a previous study done by members of our group [5,25].

### Leukocyte VEGF does not explain the VEGF release seen in stored platelets

**Figure 2:**
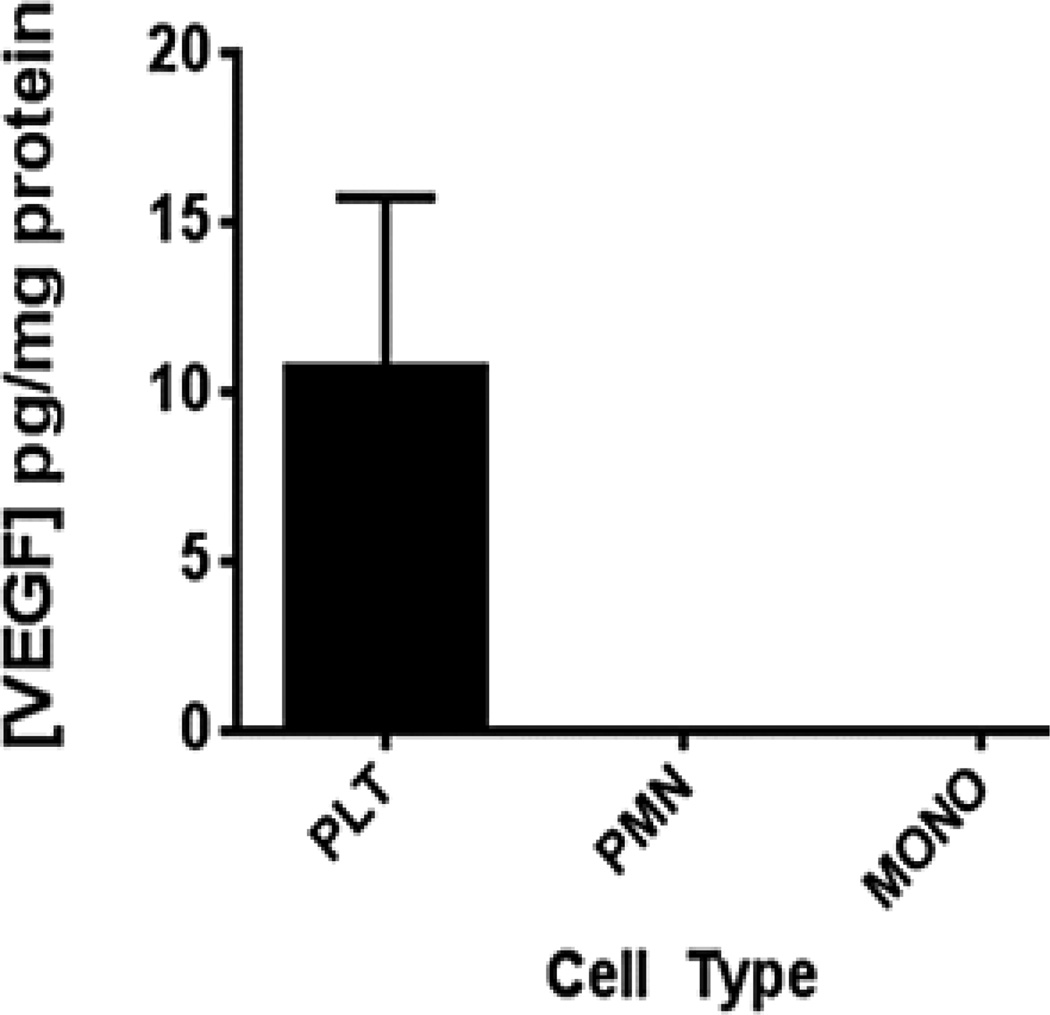
VEGF content within pellets of apheresis platelets (PLT), whole blood granulocytes (PMN), and whole blood monocytes (MONO) normalized to mg total protein, expressed as mean ± SEM. At monocyte and granulocyte concentrations 100-fold greater (2 × 10^4^ cells/ml) than the small numbers of these cells contaminating platelet units, no detectable VEGF was found in lysates. Platelet lysates (1011 cells) had substantial VEGF content. Results are depicted as group mean ± SEM

Platelet pellets from apheresis units subjected to isotonic lysis demonstrated measureable amounts of VEGF (Figure 2). Leukocyte content of the eight apheresis platelet units (201 ± 320 total leukocytes/ml; mean ± SEM) was determined to be on average 50-fold less than reported levels in pooled random donor platelets, and half that reported in another study of apheresis platelets [5]. The amount of WBC contamination (based on day 2 determinations) did not correlate with [VEGF] in these stored platelet units (r = 0.194, p = 0.75). To further evaluate the role of contaminating VEGF of leukocyte origin, lysis of leukocyte pellets obtained from 4 individual donors (separate from the apheresis donors) was carried out using leukocyte concentrations 100-fold greater than those of contaminating leukocytes from within the apheresis units. Lysis of 2 × 10^4^ mononuclear leukocytes/ml (n=4) produced no VEGF release within the detection limits of the assay (8 pg/ml), nor did lysis of 2 × 10^4^ granulocytes/ml (n=3) (Figure 2).

### Circulating VEGF at concentrations seen in apheresis platelet supernatants binds avidly and specifically to isolated perfused rat lungs

**Figure 3:**
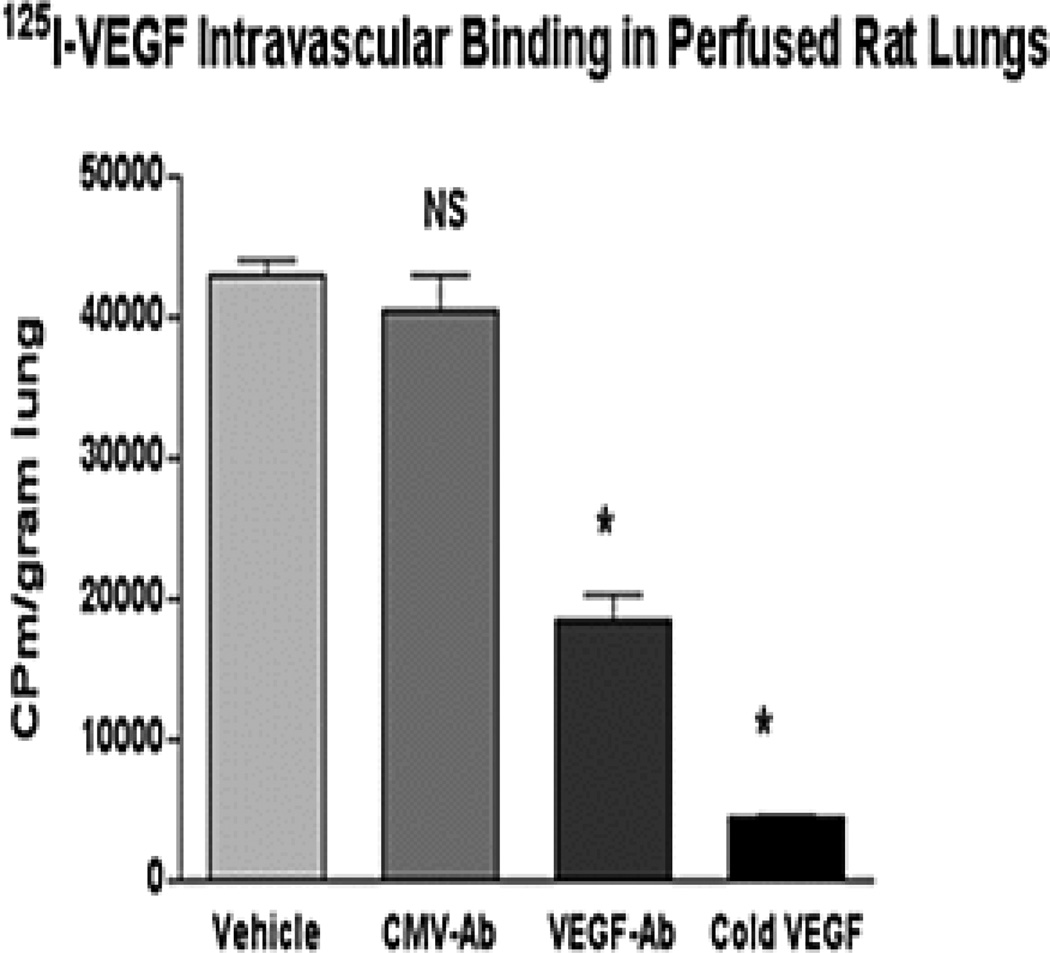
Lung binding of 125I-VEGF added to the perfusate of isolated perfused rat lungs (IPRL), as determined by counts per minute (CPM) of radioactivity per gram of lung tissue. The final [125I-VEGF] was 1 pM (23 pg/ml), similar to the [VEGF] of platelet supernatants in this study. 125I-VEGF bound avidly to VEGF receptors within the pulmonary vasculature (Vehicle, pre-incubation of 125I-VEGF with 10 ul of PBS). This binding amounted to 30–40% of added radioactivity and was specific to VEGF receptors as it was potently inhibited by pre-perfusion with a 1000-fold excess concentration of unlabeled (“Cold”) VEGF and was strongly inhibited when 125I-VEGF was pre-incubated with a VEGF antibody (VEGF-Ab in 10 ul of PBS), whereas a control antibody (CMV-Ab in 10 ul of PBS) had no significant effect. Perfusion with 125I-VEGF was done for 15 minutes. * P < 0.0001 by unpaired t-test. Results are depicted as group mean ± SEM

125I-VEGF (1 pM, or 23 pg/ml) added to the lung perfusion solution bound avidly and specifically to VEGF receptors within the pulmonary microvasculature. This 125I-VEGF binding under control conditions (PBS pre-incubation with 125I-VEGF) amounted to 30–40% of added radioactivity (about 20% of the initial radioactivity remained in perfusate at the end of experiments, the rest of the radioactivity appeared mostly absorbed onto plastic surfaces of tubing and the buffer reservoir). Binding of VEGF within hearts was minimal, being 1.81 ± 2.19% (mean ± SEM) of lung binding. Binding was specific for endothelial VEGF receptors as it was inhibited nearly completely (to 10.5% of vehicle control) by a 1000-fold (1 nM) excess concentration of unlabeled or “cold” VEGF in the perfusate (vehicle control 43,115 ± 994 cpm/gram wet lung; “cold” VEGF 4,517 ± 133 cpm/gram wet lung, P < .0001; mean ± SEM; Figure 3). Moreover, binding was further shown to be specific as it was inhibited by 55% when 125I-VEGF was pre-incubated with an anti-VEGF antibody, whereas binding was not inhibited by pre-incubation with an antibody of the same isotype that binds an unrelated target (anti-CMV antibody 40,489 ± 2,512 cpm/gram wet lung; anti-VEGF antibody 18,455 ± 1,853 cpm/gram wet lung, P < .0001; mean ± SEM; Figure 3).

The perfusion with 125I-VEGF lasted only 15 minutes, and there was little evidence that ‘cold” VEGF led to an increase in lung permeability in this uninjured model over this short time frame, since it should have led to an increase in leak of 125I-VEGF (42 kDa size) into lung tissue by bulk flow, similar to that seen with 125I-albumin leak (66 kDa size) in lung permeability models [26]. Switching to the perfusate with 1 nM unlabeled VEGF did not change mean pulmonary artery pressures – these were similar and stable during the experiment and at end-experiment between all groups (end-perfusion values before EBS/4% BSA wash: PBS control 7.4 ± 1.3 mmHg, anti-CMV antibody 7.3 ± 0.9 mmHg, anti-VEGF antibody 7.3 ± 1.2 mmHg, unlabeled “cold” VEGF perfusion 7.4 ± 1.4 mmHg; mean ± SEM).

### VEGF causes vascular leak in uninjured isolated perfused rat lungs

**Figure 4:**
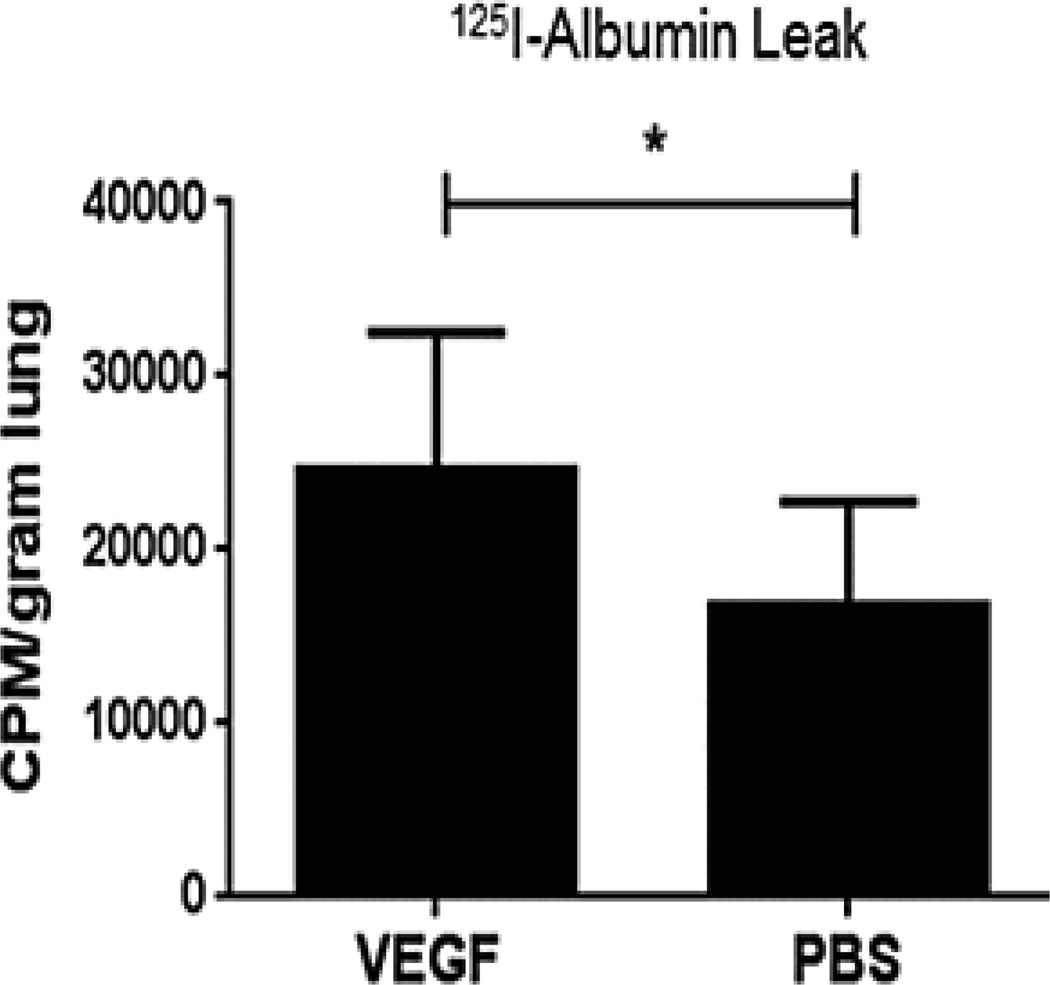
Lung leak caused by VEGF. Uninjured isolated perfused rat lungs (IPRL) were studied with 125I-albumin added to the perfusate to determine the lung vascular leak caused by unlabeled VEGF (250 pM, or 5,750 pg/ml) compared to PBS vehicle. 125I-VEGF was not used in these experiments. Leak was determined by counts per minute (CPM) of radioactivity per gram of wet lung tissue. Vascular leak with unlabeled VEGF was 1.66 ± 0.21 times that seen with PBS vehicle (mean ± SEM). Perfusion with 125I-albumin was done for 15 minutes after VEGF addition. * P < 0.05 by paired t-test

Vascular leak in uninjured isolated perfused rat lungs perfused with 125I-albumin and unlabeled VEGF at 250 pM was 1.66 ± 0.21 times that seen with PBS vehicle (mean ± SEM, p = 0.23, paired t-test, Figure 4). 125I-VEGF was not used in these experiments.

## Discussion

Non-hemolytic transfusion reactions due to platelet products are a significant source of morbidity and excessive cost; they are most often due to platelet and plasma product administration [27]. Irradiation of blood products, size filtration by gravity, and improvements in isolation techniques (such as apheresis) have all helped diminish the incidence of these reactions as they relate to leukocytes, but none of these techniques have eliminated such reactions entirely. This may be due to residual white cell contamination, donor antibodies, or due to secreted mediators. Only one specific platelet protein has been clearly associated with these reactions (soluble CD40 ligand, sCD40L) though others are known to increase during platelet storage, including RANTES [9].

These data confirm the presence of soluble VEGF in the plasma of apheresis platelet concentrates and that it increases over serial days of storage to levels substantially higher than those seen in human plasma [5]. Kluter et al found much larger elevations of soluble VEGF in pooled buffy coat-derived (non-pheresis) platelets, but VEGF levels did not change over 5 days of storage (WBC contamination was nearly 10^4^-fold higher in their study even after filtration) [6]. Edvardsen et al also examined apheresis platelet units and found similar soluble VEGF elevations as we did (with comparable low WBC contamination) but a contribution of WBC to VEGF elevations was not ruled out. We add to these investigations by excluding a role for contaminating leukocytes as the source of VEGF in our study, as no VEGF was detected in lysates of isolated monocyte or neutrophil suspensions at concentrations 100-fold greater than those contaminating our platelet products. Moreover, VEGF concentrations did not show a positive correlation with the degree of leukocyte contamination in our study. Blood products are typically transfused into peripheral veins or central veins, which was mimicked in our delivery of radiolabeled VEGF into the right ventricular outflow tract of an isolated rat heart-lung block. We found that VEGF in the range seen in the plasma fraction of our stored platelets was avidly and quickly (15 minutes) taken up by the lung vasculature. This binding was specific as it was potently blocked by unlabeled excess VEGF (which at 1 nM would block binding to VEGF receptors but not nonspecific binding to surfaces), moderately blocked by an anti-VEGF antibody, and not affected at all by a control antibody. Thus, it can be expected that the soluble VEGF in transfused platelets will be taken up preferentially by the lungs, as the lungs are the first major endothelial bed that “sees” any transfused products. Moreover, the lungs contain half of the body’s endothelial cells and the lungs are the only other organ beside the heart through which all the cardiac output must pass [28]. VEGF is a potent endothelial permeability mediator and endothelial mitogen that has been implicated in ALI pathogenesis [17,29]. VEGF receptors are mostly restricted to endothelium. Though present in many cells, VEGF is also synthesized in megakaryocytes, stored within platelets, and is present in leukocytes [30–32]. VEGF appears to act as a mediator of increased endothelial permeability in malignant effusions and in skin diseases [33]. We also found that nonradioactive VEGF perfusion caused vascular leak in the uninjured rat lung model we employed, though due to funding constraints we only tested one concentration.

The importance of this work lies in that VEGF is an effective, direct-acting mediator of endothelial permeability. The concentrations of VEGF in these platelet units are at or below concentrations where VEGF is a permeability mediator on uninjured vessels in skin, [17] moreover further amounts of VEGF may be released from transfused platelets after they engage activated/injured recipient endothelial surfaces (as seen in patients with inflammatory illnesses). Notably, the lower VEGF concentrations seen in even leuko-reduced platelet products may be relevant to leak of injured blood vessels in the “twohit” mechanism proposed for most cases of TRALI [16]. VEGF also has procoagulant properties that may contribute to the events of nonhemolytic transfusion reactions [34]. As a direct-acting permeability mediator, VEGF could also be important for the TRALI that occurs in patients with low absolute neutrophil counts, where leukocytes are neither resident in the lung nor recruitable to lung.

Our study has limitations. Others have already shown that VEGF is released during platelet storage, though changes in concentration over the storage period were not uniformly observed. We felt that a better control for WBC contamination as a potential cause of VEGF elevations was needed before proceeding with animal studies. Although we show that the potent direct acting permeability mediator VEGF increases over storage time in platelet units at a level that binds extensively to lung vessels, implicating VEGF as the predominant mediator of the lung leak and injury seen with older blood products will require further study. This is important, as VEGF reduction in platelet units using anti-VEGF antibody panning has been shown to be feasible [25]. While we showed that VEGF causes vascular leak of uninjured lungs, the concentration of VEGF we used was up to 38 times the VEGF concentration observed in day 5 platelet supernatants (it may have been less, related to VEGF binding to plastic surfaces in the perfusion apparatus). We postulate that VEGF causes leak in injured lungs at lower concentrations, consistent with the two-hit hypothesis of TRALI, and consistent with sub-picomolar VEGF effects on skin permeability [17]. More animal work will be needed to determine whether the VEGF released by platelets in storage is a main causative factor of TRALI, an amplifier of TRALI caused by other factors, or is unrelated to the lung leak of TRALI.

In conclusion, the elevations in soluble VEGF released with increasing days of platelet storage, even with leuko-reduced (apheresis) units, are at a level that can be expected to bind avidly to lung vasculature during transfusion. Further work will be needed to determine whether soluble VEGF is a key mediator of the TRALI that is more likely to occur when older platelets are transfused to susceptible hosts [35].
